# Successful Control of an Outbreak by Phenotypically Identified Extended-Spectrum Beta-Lactamase–Producing *Klebsiella pneumoniae* in a Neonatal Intensive Care Unit

**DOI:** 10.3390/antibiotics11111649

**Published:** 2022-11-18

**Authors:** Elena Priante, Chiara Minotti, Cristina Contessa, Margherita Boschetto, Paola Stano, Federico Dal Bello, Ettore De Canale, Elisabetta Lolli, Vincenzo Baldo, Eugenio Baraldi, Daniele Donà

**Affiliations:** 1 Neonatal Intensive Care Unit, Department of Women’s and Children’s Health, University Hospital of Padua, 35128 Padova, Italy; 2 Division of Pediatric Infectious Diseases, Department of Women’s and Children’s Health, University Hospital of Padua, 35128 Padova, Italy; 3 Department of Directional Hospital Management, University Hospital of Padua, 35128 Padova, Italy; 4 Infection Control Division, University Hospital of Padua, 35128 Padova, Italy; 5 Microbiology and Virology Unit, University Hospital of Padua, 35128 Padova, Italy

**Keywords:** outbreak, newborn, extended-spectrum beta-lactamase-producing *Klebsiella pneumoniae*, neonatal intensive care unit, multidrug-resistant organism

## Abstract

Background: Premature newborns represent a vulnerable population, at high risk of acquiring nosocomial infections during neonatal intensive care unit (NICU) admission. Multidrug-resistant organisms represent the greatest concern due to their intrinsic virulence and the limited therapeutic options. Resistant *Enterobacterales* are a growing threat for critically ill neonates, with increasing numbers of NICU outbreaks caused by extended-spectrum beta-lactamase (ESBL)-producing *Enterobacterales* being described. This study reports the early detection and successful control of an outbreak caused by ESBL-producing *Klebsiella pneumoniae* (ESBL-KP) in an Italian NICU in February 2021. Results: A total of 13 newborns tested positive for ESBL-KP between 2–9 February 2021, of whom four (31%) had a bloodstream infection. Two were critically ill, extremely premature newborns who died because of multiple comorbidities, and two were cured after treatment with meropenem. All other patients survived and were either discharged home or moved to other hospitals/wards in good clinical condition. ESBL-KP ST45 was found in all isolates by multilocus sequence typing (MLST) analysis. An outbreak control plan was set, including surveillance cultures for all neonates, NICU environments, and medical devices, along with the extended use of contact precautions and cohorting. In addition, the infection control plan was carried out through reinforcement and enhancement measures to guarantee maximal compliance. The outbreak was successfully controlled in seven days, given that no further cases were identified after 9 February. The source of the ESBL-KP outbreak was not identified through environmental sampling. Conclusions: Thanks to multidisciplinary management, a threatening outbreak of ESBL-KP in a NICU was controlled in few days. The prompt recognition of the event onset and the adoption of infection control interventions helped contain the bacteria spread on the ward.

## 1. Introduction

The admission of critically ill or premature infants to a neonatal intensive care unit (NICU), although life-saving, puts those infants at high risk of acquiring a nosocomial infection [1].

Gram-positive bacteria such as coagulase-negative *Staphylococci* (CoNS) and methicillin-sensitive *Staphylococcus aureus* (MSSA) are together responsible for up to 60% of late-onset sepsis (LOS) events in NICUs, as compared to Gram-negative bacteria, especially *E. coli*, *Klebsiella* spp., *Pseudomonas* spp., and *Enterobacter* spp., which are overall accountable for 18% [1,2]. Although all pathogens can be dangerous for this vulnerable population, multidrug-resistant organisms (MDRO) represent the greatest concern due to their intrinsic virulence and the limited therapeutic options [1]. It has been recently estimated that antimicrobial resistant pathogens are potentially responsible for around 30% of all global neonatal sepsis deaths [3]. Methicillin-resistant *Staphylococcus aureus* (MRSA) is the most frequently isolated resistant pathogen in the NICUs of developed countries [2]. Nonetheless, resistant *Enterobacterales* [4] represent an even more severe growing threat for critically ill neonates [5], and an increasing number of NICU outbreaks caused by extended-spectrum beta-lactamase (ESBL) producing *Enterobacterales* have been reported [6,7,8,9,10,11,12,13,14,15,16,17,18].

An outbreak in a sensitive setting such as a NICU can have tremendous consequences for affected patients. The number of NICU outbreaks reported in the medical literature are probably only a small portion of the total number of epidemic events. Nonetheless, the data presented are extremely valuable and, when aggregated, can provide a better insight into the most effective strategies to prevent and manage an outbreak event [19]. Among the infection control measures implemented during NICU outbreaks are the review of the general infection control procedures, including hand hygiene, practices for sterilization/disinfection of equipment, the preparation of infant formulae, aseptic techniques for invasive procedures, isolating/cohorting the affected patients, and personnel screening [19].

This study reports the successful and timely multidisciplinary management of an outbreak due to ESBL-producing *Klebsiella pneumoniae* (ESBL-KP) in the NICU of Padova University Hospital.

## 2. Results

The cluster counted 13 neonates (6/13 males) with heterogeneous gestational ages, body weights, and comorbidities, of whom 11 (84.6%) survived the outbreak. The main clinical features of cases are summarized in Supplementary Table S1.

The first case of positivity to ESBL-KP dates back to 2 February 2021, with isolation of the germ on mini-BAL sample (official antibiogram result on 5 February) of a term, mechanically ventilated female newborn with respiratory failure due to a Joubert Syndrome-like ciliopathy. Notably, the index case underwent a diagnostic video laryngo-tracheoscopy two weeks before the outbreak onset. No patients had reinfection. Nine (69.2%) were just colonized. Four (31%) had a bloodstream infection. Two of them were critically ill extremely premature newborns who died because of multiple comorbidities (respiratory distress syndrome requiring mechanical ventilation, postpatent ductus arteriosus closure syndrome, bronchopulmonary dysplasia, severe intraventricular hemorrhage, anemia, ascitis, late-onset sepsis and intestinal perforation, respectively; Supplementary Table S1). The remaining two were cured after meropenem treatment, but one died of other causes later. All the remaining colonized/infected patients survived and were either moved to other hospitals/wards in good clinical conditions or discharged home.

**Figure 1:**
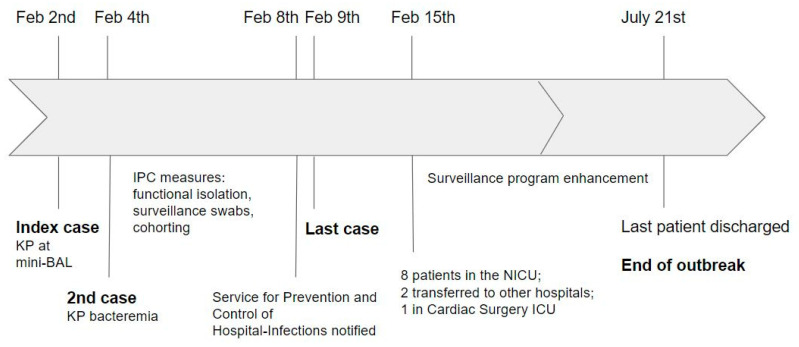
Outbreak timeline.

The time to outbreak resolution was seven days. Since 2 February, all patients on the ward underwent surveillance swabs, with the subsequent finding of six cases with positive pharyngeal swabs and three with positive blood cultures by 9 February. By 15 February, when the results of the cultures sent on 9 February were available, the total number of positive newborns increased to 13; eight were in the NICU, two moved to other hospitals, and one to the cardiosurgery intensive care unit. The last positive patient was discharged on 21 July. No further cases have been identified since mid-February (Figure 1).

Criteria for the definition of an epidemic event or a potential epidemic event are listed in Supplementary Table S2.

### 2.1. Outbreak Management

After the first case of ESBL-KP isolation on mini BAL (2 February) and the first alert notice of bacteremia in the second patient (4 February), all NICU patients were functionally isolated by contact precautions (single-use gowns and gloves). In addition, a strict microbiological surveillance program was set up utilizing pharyngeal and rectal swabs performed every 48 h on all the NICU inpatients. Early awareness of the optimal hand hygiene procedure, use of contact precautions, and disinfection of medical devices and the environment was raised among staff members. The therapeutic choices were established together with the pediatric infectious diseases consultants. The Service for Prevention and Control of Hospital Infections was promptly notified of the cluster (8–9 February), and an infection control program was implemented together with the NICU team. Cohorting was implemented with cases, close contacts and non-contacts with negative swabs being allocated in three different rooms. Great attention was placed on the adoption of functional isolation measures for each patient regardless of MDRO colonization. Active surveillance swabs were extended to all the patients in the sub-intensive ward. Culture samples were also taken from the environment, including medical devices, stethoscopes, ventilation equipment, incubators, glucometers, infusion pumps, portable ultrasound, computer keyboards, automatic door openers, milk samples and nursing carts. The ongoing outbreak was reported to all the ward/hospital destinations of transferred newborns.

### 2.2. Reinforcement and Enhancement of Infection Control Program

Since the report of a total of 13 cases in mid-February, reinforcement of correct hand hygiene techniques and contact precaution was implemented, together with periodical infrared hand scanning to check proper cleaning (Semmelweis Hand In Scan), refresh meetings, updating, and group educational seminars with a review of the literature. Contact precautions were established as mandatory for entering the wards for external consultants and parents. The initial environmental decontamination measures were enhanced. Mobile phones were forbidden in patient zones and only permitted in the common areas. The so-called “patient zones” were created in both wards and delimited around each cot by colored marks on the floor to make clear where to put on and off contact precautions before and after approaching the patient. One bed zone per room was kept empty and used as a “dressing/undressing” area. The bed number was decreased to 13 cots in the NICU and 12 in the sub-intensive ward, and pregnant inpatients in stable conditions were temporarily deviated to other sites. On 15 February, the infection control team established mandatory testing with surveillance swabs to be performed daily for negative newborns, while positive newborns were considered colonized until discharge. Rectal swab surveillance was also established for inpatient pregnant women in gynecology wards to identify possible ESBL carriers. After 19 February, a gradual opening to new admissions was possible, even maintaining an overall reduction of beds to allow the maintenance of the dressing/undressing area in each room. Since then, active twice-weekly surveillance was maintained. Cohorts of patients with MDRO colonization and contact precautions for each patient zone have been maintained after discharge of the last case, with random inspections to check adherence to the hand hygiene procedure and contact precaution adoption. An official document for internal use as a guide for infection prevention and control (IPC) was implemented by the infection control team.

### 2.3. Treatment

Colonized cases were not treated except in critical conditions to avoid the risk of selection pressure and the possible emergence of further antibiotic resistance, as recommended (http://www.salute.gov.it/imgs/C_17_pubblicazioni_2660_allegato.pdf, accessed on 2 February 2021). Intravenous vancomycin (10–15 mg/kg every 18, 12 or 8 h according to age) and meropenem (20–40 mg/kg every 12 or 8 h) in combination were the antibiotics of choice for the empirical treatment of suspected infections. Meropenem was used for sepsis/central-line-associated bloodstream infection (CLABSI) targeted treatment.

### 2.4. Antibiotic Resistance and Molecular Characteristics

Sequences of seven housekeeping genes were obtained for all the *K. pneumoniae* outbreak strains. A dominant strain, sequence type (ST) 45, was found (13/13 cases, Table 1). Our results showed that all ESBL-KP strains were resistant to amoxicillin-clavulanic acid and cephalosporins (except ceftazidime/avibactam and ceftolozane/tazobactam) and susceptible to meropenem, imipenem, and colistin (details shown in Table 1).

### 2.5. Environmental and Staff Surveillance

Samples of human and formula milk were not found to be contaminated. Samples were collected from floors, roofs, walls, air, water, and doors, and also in common spaces and all the equipment surfaces (cots, circuits, etc.), according to the protocol proposed by our infection control team. No positivity was found in environmental cultures, even though mixed microbial flora (*Staphylococcus* and *Micrococcus* spp.) was found on various surfaces. All the staff underwent nasal and rectal swabs, which all tested negative. Hands underwent a periodical infrared scan after washing. All the staff was trained and passed the tests.

## 3. Materials and Methods

### 3.1. Study Design, Setting, and Population

This is a retrospective observational study. An ESBL-KP outbreak took place in the III level 20-bed NICU of Padova University Hospital at the beginning of February 2021, counting three six-bed rooms (T1, T2, T3) and two single isolation rooms (I1 and I2). Later, the 15-bed sub-intensive neonatal ward was also involved. The staff comprises 12 neonatology consultants, 20 pediatrics residents, 48 nurses, and four social health workers. The neonatal population at the time of the outbreak mainly included preterm and extremely preterm infants, but also neonates affected by congenital malformations or other comorbidities requiring intensive care. Before the outbreak, surveillance swabs were not performed routinely, but only as screening on admission. For this reason, the incidence of previous ESBL-KP colonization cases was not available. No cases of ESBL-KP infection were registered in 2020.

### 3.2. Case Definition and Inclusion Criteria

A case was defined as a newborn with either infection or colonization by ESBL-KP. Infection was defined by positive blood, cerebrospinal fluid, urine, or bronchoalveolar lavage (BAL) cultures with symptoms. Colonization was defined as the positivity of surveillance swabs without clinical symptoms. Data from medical records of all newborns admitted to the NICU of the Department of Women’s and Children’s Health of the University Hospital of Padova (Veneto Region, Italy) between January 2021 and December 2021, with either infection or colonization by ESBL-KP, were included.

### 3.3. Data Collection

For each patient, clinical, demographic, diagnostic, and prescription data were manually collected from electronic medical records. Privacy was guaranteed by assigning to each patient a unique, anonymous study code, and not collecting personally identifying data. The investigations were carried out following the rules of the Declaration of Helsinki of 1975 (https://www.wma.net/what-we-do/medical-ethics/declaration-of-helsinki/, last accessed on 30 September 2022), revised in 2013.

The following variables, selected a priori, were evaluated: sex, gestational age (GA), post-natal age (PNA), birth weight (BW), comorbidities, admission room, invasive procedures, infection type (early or late onset sepsis (EOS/LOS), pneumonia, central line associated bloodstream infection (CLABSI)) vs. colonization, antibiotic regimen, antibiotic resistance and molecular typing analysis of ESBL-KP isolates, admission and discharge dates, outcome (survival/exitus).

### 3.4. Outcomes

The primary outcomes are survival vs. mortality of patients with ESBL-KP infection or colonization and time to outbreak resolution. Secondary outcomes include description of multilocus sequence typing (MLST) ESBL-KP of isolates and antimicrobic susceptibility testing, and description of the management of patients with ESBL-KP positivity with infection control program measures and environmental surveillance.

### 3.5. Outbreak Management Strategies and Treatment

Among the employed strategies to contain the outbreak were the use of contact precautions, such as disposable gloves and gowns, cohorting, active surveillance swabs of patients, staff and the environment, awareness and retraining of the optimal hand hygiene procedure, and disinfection of devices and surfaces.

The empirical combination of vancomycin and meropenem was the treatment of choice in colonized patients who presented signs/symptoms of infection. The treatment was de-escalated to only meropenem in case of a microbiologically confirmed invasive infection due to ESBL-KP.

### 3.6. Identification of ESBL Isolates in Cultures and Antimicrobial Susceptibility Testing

All surveillance swabs were cultured on CHROMagar ESBL (MEUS SRL, Padova, Italy), a selective chromogenic screening medium for the isolation of ESBL-producing *Enterobacterales*. Cultures were incubated for 18–24 h at 37 °C. Identification of the species level and antimicrobial susceptibility testing of the isolates was performed by the VITEK MS and VITEK 2 automated system (BioMérieux, Grassina, Italia, S.p.A.), respectively, according to the interpretive criteria of the European Committee on Antimicrobial Susceptibility Testing. *Enterobacterales* isolates resistant to any indicator cephalosporin (ceftazidime, cefotaxime, cefpodoxime) but susceptible to carbapenems in the susceptibility testing underwent phenotypic confirmation of the ESBL production by using the E-test^®^ method on Mueller–Hinton agar. The E-test^®^ (BioMérieux, Marcy l’Étoile, France) is a gradient method based on two-sided strips containing gradients of one of the cephalosporins cefotaxime (CT), or ceftazidime (TZ) or cefepime (PM) on one end and a gradient of cephalosporin combined with clavulanic acid (4 μg/mL fixed concentration) on the other end, intended to confirm the presence of ESBL enzymes, inhibited by a β-lactamase inhibitor such as clavulanic acid, in *Enterobacterales*. Interpretation criteria followed the manufacturer’s recommendations, and the test was considered positive for ESBL if a greater than or equal to eightfold reduction was observed in the minimum inhibitory concentration (MIC) of TZ, CTX, and/or PM combined with clavulanic acid compared with the MIC of the TZ, CTX and/or PM without clavulanic acid or if a phantom zone was present.

Antimicrobial susceptibilities are interpreted according to EUCAST breakpoints as updated in breakpoint tables for interpretation of MICs and zone diameters (Version 11.0, valid since 1 January 2021).

### 3.7. Genotyping Analysis

Isolated colonies of each *K. pneumoniae* strain were emulsified in 350 µL of sterile water. Then, heat shock was used to extract DNA from *K. pneumoniae* colonies, which can be utilized in performing PCR and DNA sequencing. The bacteria were heated at 99 °C for 30 min in a water bath, centrifuged at 13,500 rpm for 5 min and 1 µL of supernatants was transferred to a new microcentrifuge tube containing the PCR mix (15 µL of RedTaq Readymix (Sigma-Aldrich, St. Louis, MO, USA), 15 µL of sterile water, and 1 uL of 10 uM primer). Multilocus sequence typing (MLST) by using seven genes (gapA, infB, mdh, pgi, phoE, rpoB, and tonB) was performed for all isolates tested according to the protocol (with universal sequencing primers) described on the *K. pneumoniae* MLST website (https://bigsdb.pasteur.fr/klebsiella/primers-used/, accessed on 9 February 2021).

Sequence types (STs) were assigned by using the MLST database on the Pasteur Institute MLST website (https://bigsdb.pasteur.fr/cgi-bin/bigsdb/bigsdb.pl?db=pubmlst_klebsiella_seqdef, accessed on 15 February 2021).

## 4. Discussion

In recent years, an alarming trend toward high levels of resistant bacteria colonizing NICU infants has been observed globally [5]. In Italy in particular, the antimicrobial resistance burden is of great concern with a high prevalence of resistant bacterial isolates, significantly above the European Union average (http://www.salute.gov.it/imgs/C_17_pubblicazioni_2660_allegato.pdf, accessed on 30 September 2022). Critically ill infants requiring intensive care, especially those born prematurely, are invariably at high risk of exposure to bacterial infections, one of the leading causes of infant mortality and morbidity. Preterm babies are highly vulnerable for many reasons: immunological immaturity, ineffective skin and mucosal barrier, need for prolonged antibiotic treatments and use of invasive devices [20]. They are often cared for in crowded conditions that make them more prone not only to invasive disease at an individual level, but also to the dissemination of resistant bacteria into the environment, potentially triggering dangerous outbreaks. In addition, the colonized infants represent a potential source of MDRO dissemination into the community, also after discharge [21]. Remarkably, it has been reported that colonization by MDRO might persist up to 2–5 years after NICU discharge, thus further highlighting its impact on public health [22]. Other important aspects to be considered are the consequences of a sustained lack of timely de-escalation of broad-spectrum, empirical antibiotic regimens, which contribute to the instauration of antimicrobial resistance. De-escalation practices should be encouraged in stable patients without risk factors [23], as soon as the etiologic agent is identified, to avoid selection pressure.

This study describes the rapid and successful management of an outbreak caused by ESBL-KP. The prompt recognition of the event onset and the adoption of control interventions helped contain the bacteria spread on the ward. The outbreak was controlled within seven days, which is a considerably shorter time as compared to what is described in the literature [17,18]. Since 2 February, positive cultures were found in other 12 patients by 9 February, with no further cases at the surveillance program (Figure 1). Thus, although the outbreak was declared to end on 21 July when the last positive patient was discharged, it was effectively controlled in seven days, different from several reports from the literature, in which outbreak control was reached within weeks or months. This result was achieved through various integrated actions, including active microbiological surveillance, cohorting, developing a multidisciplinary team, implementing IPC procedures, and a modest reduction of cots. The role of routine surveillance swabs in preventing outbreaks is, however, controversial [24,25].

The first and probably most important challenge when dealing with an outbreak is the timely identification of its onset. A pragmatic and shared definition of when an outbreak might be considered as such in the setting of the NICU is quite challenging. The CDC stated that an “epidemic refers to an increase, often sudden, in the number of cases of a disease above what is normally expected in that population in that area.” An outbreak carries the same definition of an epidemic but is often used for a more limited geographic area” [26]. The timely recognition of the onset of a potential outbreak is crucial for the prompt setup of the appropriate control procedures [27,28]. Remarkably, some ESBL-KP outbreak reports show how retrospective analysis often revealed the circulation of the microorganism a long time before the alert for the outbreak, also in NICUs where microbiological surveillance was already in place [12,15].

Hence, every NICU should make efforts to set up its criteria to generate an “alarm” for possible epidemic events, considering international recommendations and local epidemiology and setting. Based on definitions proposed by the English Department of Health and Antimicrobial Resistance and Healthcare-Associated Infection committee [26] as well as those proposed by Decembrino and colleagues [29], a list of criteria and scenarios indicative of a possible epidemic event, applicable to this setting, have been identified (Supplementary Table S2). In most laboratories, the screening procedure consists of surveillance swab cultures on a selective screening medium, followed by species identification and susceptibility testing. The turnaround time can vary from 1 day (for negative cultures) to 4 days (in the case of positive cultures). To implement infection control promptly, a rapid molecular screening by real-time polymerase chain reaction should be adopted in the future identification of a possible outbreak onset [30,31].

Although the spread of the microorganism was rapidly stopped in the NICU, the burden related to the epidemic event was relevant, with five patients presenting infection (three CLABSI, one septic shock and one VAP). The dramatic consequences of outbreaks due to MDRO in NICUs, particularly resistant *Enterobacterales*, are well known. In a review published by Stapleton et al., a mortality rate of 16% is reported, in which the denominator is the total number of neonates infected by the microorganism. Notably, *Klebsiella* spp. were the most frequently isolated *Enterobacterales* in this review [16].

The strains isolated in the NICU ward belonged to sequence type 45 when analyzed by MLST, a method able to distinguish strains based on DNA sequences of internal fragments of bacteria [31]. The spread of this strain in neonatal healthcare facilities has been previously reported; in a study performed in a tertiary hospital in Tanzania, among ESBL-producing *Enterobacterales*, *Klebsiella pneumoniae* ST45 was the predominant cause of neonatal sepsis and mortality as well as colonization [32].

Concerns about ESBL-KP are related to the limited therapeutic options and the evidence that resistant strains are more virulent. ESBL production is known to be associated with a greater expression of virulence factors, including biofilm formation ability, cell invasion proteins and fimbrial adhesins synthesis, siderophores production, and hyper-mucoviscosity [8,9,10,11,12,13].

The identification of the source of nosocomial outbreaks often proves difficult. It is estimated that up to half of the investigations for the origin of the outbreaks gave no results, leaving the cause unidentified [19,33]. Environmental samplings failed to find the source of the outbreak, as in our case. In the review published by Stapleton et al., the outbreak’s source was identified in 43% of the analyzed outbreak reports. Notably, in 11% of the studies, the index case was identified, and in similar percentages, the source of infection turned out to be the equipment/environment (including IV fluids, breast milk, feed and cleaning solutions) or healthcare workers (HCWs) [16]. Outbreaks from environmental contamination (mostly breast milk) are also reported by other authors, like Rettedal and colleagues [34]. Interestingly, our index case underwent a diagnostic video laryngo-tracheoscopy two weeks before the onset of the outbreak. Numerous reports describe outbreaks associated with device contamination, particularly endoscopes [35]. Among the multiple virulence factors that permit the environmental persistence of *K. pneumoniae*, type 1 and 3 fimbriae permit adherence to abiotic surfaces. In addition, *K. pneumoniae* can form biofilms by using a self-produced matrix of an extracellular polymeric substance comprising polysaccharides, proteins, and DNA [36]. These factors, in addition to facilitating the adherence and persistence on biotic and abiotic surfaces, might affect the sensitivity of sampling methods [35,36].

A set of measures to control the spread of the microorganism were performed in our ward simultaneously, so that it is not possible to identify the single contribution of each action. The reinforcement of correct hand hygiene procedures, the introduction of contact precautions for all patients, cohorting and creating a “patient zone” were probably the most effective measures, enabling only a modest occupancy reduction without needing to close the ward. The creation of a multidisciplinary team was another key element for success, as already demonstrated in previous reports [18,37]. In addition, planning regular education and updating meetings with all HCWs is another strategy that has proved to be effective for implementing IPC practices [1].

Nonetheless, HCWs invariably face increased emotional, physical and mental stress during an outbreak event. For instance, the isolation of patients and the use of protective equipment might result in social and psychological side effects, possibly leading to impaired quality of care. Although not addressed and measured in our setting, this issue probably deserves further exploration. Only a few reports have evaluated the emotional response of HCWs to epidemic events. Bushuven et al. have reported the main perceptions of HCWs concerning MDRO management. The first theme that emerged is that HCWs felt a significant gap in knowledge on MDRO, which might lead to confusion, uncertainty, and underestimation of risks related to inadequate measures. Secondly, the author found out that anxiety and anger were the main emotional effects experienced by HCWs [38]. Given these observations, it has to be highlighted how education on IPC cannot be confined to the simple transmission of a list of hygiene measures to be observed, but should rather rely on knowledge transfer and include reflection moments in which the perspective, attitudes, and emotions of professionals can get out. Many IPC interventions are relatively low-cost and straightforward [39].

Nonetheless, infection control measures often fail to be implemented and maintained, as this requires behavioral changes to ensure adequate adherence [40]. In this perspective, implementation science, as the study of methods that promote the adoption of evidence-based practice into healthcare use, may help determine the facilitators and barriers to successfully incorporating IPC measures [41]. Implementation science not only aims at understanding things that work but how and why they work, which is a key element to ensuring that best practices can be successfully adopted.

In conclusion, MDRO outbreaks represent dramatic events, which, in addition to having a detrimental clinical impact, are associated with public health, economic, and legal issues. All the stakeholders involved in IPC interventions should make every effort to develop shared and homogeneous definitions, actions, and effectiveness measurements to prevent, promptly detect and/or quickly eradicate any possible outbreak event.

## Figures and Tables

**Table 1:** Results of multilocus sequence typing (MLST) using seven genes (gapA, infB, mdh, pgi, phoE, rpoB, and tonB).

Patient	MLST Scheme	ST Clone	Standard Housekeeping Loci							Type of Specimen	Antimicrobial Resistance Phenotype
			gapA	infB	mdh	pgi	phoE	rpoB	tonB		
1	*K. pneumoniae*	ST 45	2	1	1	6	7	1	12	Mini-BAL, pharyngeal swab	Amoxicillin-clavulanic acid, cephalosporins (except ceftazidime/avibactam and ceftolozane/tazobactam), ciprofloxacin, gentamicin, trimethoprim/sulphamethoxazole, tobramycin
2										Blood culture, pharyngeal swab	Amoxicillin-clavulanic acid, cephalosporins (except ceftazidime/avibactam and ceftolozane/tazobactam), ciprofloxacin, gentamicin, trimethoprim/sulphamethoxazole, tobramycin
3										Blood culture, pharyngeal swab	Amoxicillin-clavulanic acid, cephalosporins (except ceftazidime/avibactam and ceftolozane/tazobactam), ciprofloxacin, gentamicin, trimethoprim/sulphamethoxazole, tobramycin, Piperacillin/tazobactam
4										Cutaneous and pharyngeal swab, blood culture	Amoxicillin-clavulanic acid, cephalosporins (except ceftazidime/avibactam and ceftolozane/tazobactam), ciprofloxacin, gentamicin, trimethoprim/sulphamethoxazole, tobramycin
5										Pharyngeal swab, blood culture	Amoxicillin-clavulanic acid, cephalosporins (except ceftazidime/avibactam and ceftolozane/tazobactam), ciprofloxacin, gentamicin, trimethoprim/sulphamethoxazole, tobramycin
6										Pharyngeal swab	Amoxicillin-clavulanic acid, cephalosporins (except ceftazidime/avibactam and ceftolozane/tazobactam), ciprofloxacin, gentamicin, trimethoprim/sulphamethoxazole, tobramycin
7										Rectal swab	Amoxicillin-clavulanic acid, cephalosporins (except ceftazidime/avibactam and ceftolozane/tazobactam), ciprofloxacin, gentamicin, trimethoprim/sulphamethoxazole, tobramycin, piperacillin/tazobactam, amikacin
8										Rectal swab	Amoxicillin-clavulanic acid, cephalosporins (except ceftazidime/avibactam and ceftolozane/tazobactam), ciprofloxacin, gentamicin, trimethoprim/sulphamethoxazole, tobramycin, piperacillin/tazobactam, amikacin
9										Rectal and pharyngeal swab	Amoxicillin-clavulanic acid, cephalosporins (except ceftazidime/avibactam and ceftolozane/tazobactam), ciprofloxacin, gentamicin, trimethoprim/sulphamethoxazole, tobramycin
10										Rectal swab	Amoxicillin-clavulanic acid, cephalosporins (except ceftazidime/avibactam and ceftolozane/tazobactam), gentamicin, trimethoprim/sulphamethoxazole, tobramycin
11										Rectal and pharyngeal swab	Amoxicillin-clavulanic acid, cephalosporins (except ceftazidime/avibactam and ceftolozane/tazobactam), ciprofloxacin, gentamicin, trimethoprim/sulphamethoxazole, tobramycin
12										Rectal swab	Amoxicillin-clavulanic acid, cephalosporins (except ceftazidime/avibactam and ceftolozane/tazobactam), gentamicin, trimethoprim/sulphamethoxazole, tobramycin
13										Rectal swab	Amoxicillin-clavulanic acid, cephalosporins (except ceftazidime/avibactam and ceftolozane/tazobactam), gentamicin, trimethoprim/sulphamethoxazole, tobramycin

Legend: MLST, multilocus sequence typing; ST, sequence type; *K. pneumoniae*, *Klebsiella pneumoniae*; mini-BAL, mini-bronchoalveolar lavage.

## Data Availability

The data presented in this study are available in the article (Table 1) and Supplementary Materials.

## Supplementary Materials

The following supporting information can be downloaded at: https://www.mdpi.com/article/10.3390/antibiotics11111649/s1, Table S1. Clinical characteristics of patients infected or colonized with ESBL-KP during the outbreak; Table S2: Criteria for definition of an epidemic event or a potential epidemic event.
