# Supplementary material for: Successful Control of an Outbreak by Phenotypically Identified Extended-Spectrum Beta-Lactamase–Producing Klebsiella pneumoniae in a Neonatal Intensive Care Unit
Source: Antibiotics (Basel). 2022 Nov 18;11(11):1649. doi: 10.3390/antibiotics11111649 (PMC9686647; doi:10.3390/antibiotics11111649)
Supplement: Supplementary file 1 [file antibiotics-11-01649-s001.zip › antibiotics-1989260-supplementary.pdf]

Supplementary Table S1. Clinical characteristics of patients infected or colonized with ESBL-KP during the outbreak

Case	*Sex*	*Main morbidities*	*BW(g)*	*Room*	*Invasive procedures*	*Infection type*	*Positive culture sample (date)*	*Antibiotic regimen*	*Outcome*
1	F	Ciliopathy - Joubert syndrome like<br>Bone Dysplasia<br>Respiratory failure	3430	T2	UVC<br>CVC mechanical ventilation<br>LTS	VAP	mini-BAL (02/02)<br>Pharyngeal swab (05/02)	Meropenem + vancomycin, then meropenem	Cured, later dead (for other causes)
2	M	Extremely preterm (24 gws)<br>RDS<br>BPD	700	T1	UVC<br>UAC<br>ECC<br>non-invasive ventilation	LOS (CLABSI)	Blood culture (02/02)<br>Pharyngeal swab (05/02)	Vancomycin + amikacin, then amikacin + meropenem, then meropenem	Cured, discharged
3	M	Moderate preterm (31+4 gws)<br>IUGR<br>Suspected syndrome (esophageal atresia, CHD)<br>RDS<br>Pneumothorax	1200	T1	UVC<br>UAC<br>ECC<br>mechanical ventilation<br>pleural drainage<br>LTS	LOS (CLABSI)	Blood culture (03/02)<br>Pharyngeal swab (05/02)	Vancomycin + meropenem, then meropenem	Cured, later dead (for other causes)

4	F	Extremely preterm (23+1 gws)<br>IUGR<br>RDS<br>Patent ductus arteriosus<br>IVH II	400	T1	UVC<br>UAC<br>ECC<br>mechanical ventilation<br>Cardiac surgery	Colonized, then septic shock	Cutaneous swab (03/02),<br>Pharyngeal swab (05/02)<br>Blood culture (09/02)	Vancomycin + meropenem, then meropenem	Dead
5	M	Extremely preterm (24 gws)<br>Severe RDS<br>Pulmonary hypertension<br>Cardiac dysfunction with systemic hypotension<br>Patent ductus arteriosus<br>IVH III and periventricular hemorrhagic infarction<br>Bowel perforation	730	T3	UVC<br>ECC<br>mechanical ventilation	Colonized, then LOS (CLABSI)	Pharyngeal swab (06/02)<br>Blood culture (07/02)	Vancomycin+ meropenem	Dead (palliative care)
6	F	Late preterm (36+5 gws)<br>IUGR<br>HIE	1925	T1	UVC<br>UAC<br>non-invasive ventilation	Colonized	Pharyngeal swab (05/02)	Not treated	Moved to another hospital

7	M	Moderate preterm (31+4 gws)<br>RDS	1620	T3	UVC<br>non-invasive ventilation	Colonized	Rectal swab (08/02, on admission in another hospital)	Not treated	Moved to another hospital
8	F	CHD	3800	T2	UVC<br>CVC	Colonized	Rectal swab (09/02)	Not treated	Moved to another ward
9	F	Late preterm (35+1 gws)<br>IUGR<br>Down syndrome<br>CHD<br>Respiratory failure<br>NEC	1660	Sub-intensive ward – room 2	CVCs<br>Mechanical ventilation<br>Abdominal surgery<br>Cardiac surgery	Colonized	Rectal and pharyngeal swab (09/02)	Not treated	Moved to another ward
10	M	Moderate preterm (31+4 gws)<br>RDS	1450	I	UVC	Colonized	Rectal swab (09/02)	Not treated	Discharged
11	F	Moderate preterm (32 gws)<br>Alagille syndrome<br>CHD<br>Pleural effusion	1865	Sub-intensive ward – room 4	UVC<br>ECC<br>Mechanical ventilation	Colonized	Rectal and pharyngeal swab (09/02)	Not treated	Moved to another hospital

12	F	Extremely preterm (27+4)<br>RDS	1160	Sub-intensive ward – room 2	UVC<br>non-invasive ventilation	Colonized	Rectal swab<br>(09/02)	Not treated	Discharged
13	M	Moderate preterm (30)<br>RDS<br>Patent ductus arteriosus	1600	Sub-intensive ward – room 4	UVC<br>non-invasive ventilation	Colonized	Rectal swab<br>(09/02)	Not treated	Discharged

Legend: BW: birth weight; F: female; M: male; gws: gestational weeks; RDS: respiratory distress syndrome; BPD: bronchopulmonary dysplasia; IUGR: intrauterine growth restriction; CHD: congenital heart disease; NEC: necrotizing enterocolitis; IVH: intraventricular hemorrhage HIE: hypoxic-ischemic encephalopathy; T1-3: NICU six-bed rooms; I: isolation room; UVC umbilical venous catheter; UAC: umbilical arterial catheter; CVC: central venous catheter; ECC: epicutaneous-caval catheter; LTS: laryngotracheoscopy; VAP: ventilator-associated pneumonia; LOS: late-onset sepsis, CLABSI: central-line-associated bloodstream infection; BAL: bronchoalveolar lavage.

• A case of systemic infection by ESBL-PE, CPE or CRE
• A case of systemic infection by *Pseudomonas Aeruginosa*
• A case of systemic infection by an unusual gram-negative bacteria (e.g. *Citrobacter* spp, *Serratia* spp, *Acinetobacter baumannii*, *Burkholderia* spp, *Stenotrophomonas maltophilia*)
• 2 or more isolates of the same microorganism (except CoNS) from a sterile site in the last 30 days
• 2 or more isolates of the same MDRO or of the same unusual gram-negative from a non-sterile site in the last 30 days

Supplementary Table S2: Criteria for definition of an epidemic event or a potential epidemic event

Legend: ESBL-PE: extended-spectrum beta-lactamase-producing *Enterobacterales*; CPE: carbapenemase-producing *Enterobacterales*; CPE: carbapenem-resistant *Enterobacterales*; CoNS: Coagulase-negative *staphylococci*; MDRO: multidrug-resistant organism
